# Less is more. Exploring opportunities and challenges of digital crowdsourcing for political parties

**DOI:** 10.12688/openreseurope.22121.2

**Published:** 2026-04-16

**Authors:** Francseco Nasi

**Affiliations:** 1 Universita degli Studi di Bologna Dipartimento di Scienze Politiche e Sociali, Bologna, Emilia-Romagna, Italy

**Keywords:** Political parties, digital crowdsourcing, internal party democracy, deliberation, digital platforms, democratic innovations

## Abstract

**Background:**

Political parties across liberal democracies face a persistent crisis of legitimacy, representation, and membership. In response, scholars and practitioners proposed a range of deliberative reforms aimed at making parties more internally democratic. Yet such innovations have proven difficult to implement due to structural features inherent to political parties, including hierarchical organization and electoral imperatives. Similarly, digital platforms promised to revolutionize internal democracy but largely disappointed expectations. This impasse highlights the need for lighter forms of democratic engagement that may better align with the operational realities of parties. Among these alternatives, digital crowdsourcing emerges as a possible path forward. Digital crowdsourcing refers to processes in which organizations use technology to tap into people’s distributed knowledge, combining bottom-up input with top-down coordination to solve problems, carry out tasks, or generate ideas.

**Methods:**

This theoretical paper develops an analytical framework tailored to the organizational and democratic specificities of political parties. I propose a typology of digital crowdsourcing for parties based on two dimensions (policy impact and power structure) yielding four forms: vertical, performative, expressive, and democratic crowdsourcing.

**Results:**

Thanks to this typology, I identify three core opportunities associated with the adoption of these tools: enhanced democratic participation, increased flexibility, and improved accessibility for members and supporters. Conversely, I outline three central challenges: tensions between inclusion and exclusion, risks of elite capture, and conflicts between competing sources of democratic legitimacy. Finally, I present a set of strategies for achieving a feasible democratic crowdsourcing in political parties.

**Conclusion:**

Integrating digital democratic innovations into political parties (especially long-established ones) remains particularly challenging. However, lighter forms of participation, such as digital crowdsourcing, may be more feasible to implement.

## Introduction

Political parties are widely regarded as one of the vital institutions for the functioning of liberal democracy. As Schattschneider (1942) ^1^ claimed, democracy is unthinkable save in terms of political parties. Yet, they are also the least trusted and most vituperated body of democratic life (Rosenblum, 2009). ^2^ According to the World Values Survey (2017–2022 wave), across G7 countries, an average of 79.9% of the population expresses little or no trust in political parties (EVS/WVS, 2024). ^3^ The crisis of political parties is a long-standing and complex issue that cannot be fully explored within the scope of this paper (Mair, 2013). ^4^ It involves not only a decline in public trust but also a shrinking number of individuals willing or interested in participating in party life (Dalton & Wattenberg, 2002), ^5^ as well as a diminished capacity of parties to deliver on their promises and to effect meaningful change in citizens’ lives (Crouch, 2002). ^6^

In recent years, a growing body of scholarship (and, to a lesser extent, a corresponding shift in political practice) explored how political parties might change to reverse their declining trust and legitimacy. From an academic perspective, some contributions emphasized the need to strengthen the deliberative dimension of political parties. Invernizzi Accetti and Wolkenstein (2017, p. 1), ^7^ for example, argue that “deliberative reforms are the most appropriate response to the demands of an increasingly more cognitively mobilized citizenry which seeks self-expression and non-hierarchical forms of political engagement.”

A deliberative approach to party democracy focuses on inclusion, equality of all members, and rational discourse rather than mere preference aggregation or voting for primary candidates. However, parties who attempted to implement these principles encountered several challenges, often failing to achieve the expected positive outcomes (Gherghina, 2024) ^8^ Deliberative democracy may still be, in some respects, too demanding for political parties, which, due to their hierarchical decision-making structures, struggle to find a balance with these new instruments: a balance between the equality of all members and the necessity of a clear political line, or between the time needed for rational discussion and the fast-paced timing of contemporary “fast politics” (Rastrilla *et al.*, 2023). ^9^ Might there be space for forms of political participation that are lighter, more adaptable to the needs of political parties and able to take into account increasingly diverse forms of memberships (Scarrow, 2015)? ^10^

A promising example of a lighter form of political participation is digital crowdsourcing. Crowdsourcing *“*is an open call for anybody to participate in a task open online, where ‘the crowd’ refers to an undefined group of people who participate” (Aitamurto, 2012, p. 8). ^11^ At odds with deliberative-oriented democratic innovations, crowdsourcing does not require any deliberative dimension (in terms, for instance, of rational discussion). It primarily involves collecting citizens’ opinions, feedbacks, or creative contributions to orient public decisions and policymaking. While different models of crowdsourcing exist – such as virtual labor markets or tournament crowdsourcing – the bulk of attention and practical use focused on the open collaboration model, according to which “crowds voluntarily engage with the problems/opportunities posted by organizations through IT platforms without expectation of monetary compensation” (Taeihagh, 2017, p. 632). ^12^

This paper examines how and to what extent crowdsourcing can serve as a valuable tool for political parties, particularly in light of the challenges that have emerged in recent years about deliberative mechanisms and digital platforms. In order to do this, I propose a typology of digital crowdsourcing for political parties based on two dimensions: policy impact and power structure. This typology includes *vertical, performative, expressive*, and *democratic crowdsourcing.* Then, I identify three opportunities for political parties adopting this technology: enhanced democratic participation, increased flexibility, and improved accessibility. Conversely, I highlight three challenges: the tension between inclusion and exclusion, the risk of elite capture, and conflict between competing sources of democratic legitimacy. Finally, I outline some strategies for achieving a feasible democratic crowdsourcing.

## Internal party democracy, deliberation, and digital platforms

To better situate the potentiality of digital crowdsourcing for political parties, we firstly need to look at the debate on party change and party democratization. As Harmel and Janda (1994) ^13^ argued, party change largely rests on party goals. Building on Panebianco (1988), ^14^ they suggest that change (given the conservative natures of parties themselves) generally depends on external shocks, and these shocks are dependent on party primary goals. For vote maximizers, such shocks consist in electoral defeat or a substantial loss of popular support. For office maximizers, they are events “directly related to participation in government” (Harmel & Janda, 1994, p. 270). ^13^ For policy-seeking parties, the most consequential shocks are those that shake the party’s ideological and identity foundations (as was the fall of the Berlin Wall and the end of Soviet communism for Western communist parties). Finally, for intraparty democracy maximizers, the most significant shocks concern changes in party membership—whether in size or in its social composition—as well as broader societal transformations that “change expectations or resources with regard to participation” (Harmel & Janda, 1994, p. 271), ^13^ such as the growing demand for new forms of engagement in a digitalized environment may be.

Concerning change in the direction of intra-party democracy (IDP), the topic has been mainly dealt with by scholars analyzing mechanisms such as internal referenda and party primaries (Cross & Katz, 2013) ^15^ (Cross & Pilet, 2015) ^16^ (Rahat & Shapira, 2017). ^17^ While it is true that in some instances primaries have enabled underdogs to emerge (such as Zohran Mamdani in the New York mayoral primaries in 2025 or Elly Schlein in the Italian Democratic Party leadership contest in 2023) in many cases the democratization of the process has been more formal than substantive (Hopkin, 2001). ^18^

In recent years, scholars have focused on deeper forms of political participation, drawing inspiration from Habermas’s theory of deliberation (Habermas, 1996). ^19^ According to this theory, when political actors engage in discussions about policy issues, they should be guided by a “communicative rationality” aimed at reaching mutual understanding rather than pursuing strategic advantage or entering into a win–lose dynamic. Accordingly, arguments ought to rely on the force of the better reason, rather than on power, pressure, or coercion. This conception of democracy also places strong emphasis on normative values, including inclusivity, equality, truthfulness, and reciprocity.

Invernizzi-Arcetti and Wolkenstein (2017) ^7^ saw three primary motivations for adopting a deliberative approach to internal party democracy: providing a new incentive for cognitively mobilized participation, having a tool for shaping individual preferences, and overcoming the limits of an aggregative conception of intra-party democracy, such as party primaries. To do this practically, they suggested empowering local party branches, reforming the organizational structure of the party around the concept of representation, introducing function-specific for intra-party deliberation, and using new technologies. Gherghina *et al.* (2023) ^20^ argue that parties can promote deliberation primarily by involving members and citizens in formulating political programs or, less commonly, in leadership selection processes prior to primaries. These initiatives tend to pursue either strategic goals (linked to voting, office, or policy-seeking) or normative aims rooted in the party’s civic function as a space for participation, representation, and political socialization, with deliberation valued both intrinsically and instrumentally.

Deliberative proposals have so far encountered significant challenges in practice. Traditionally, scholars have been skeptical about the deliberative potential of political parties, given their hierarchical structure and focus on electoral competition (Cohen, 2003) ^21^ (Chambers, 2003). ^22^ Recent empirical research further underscores the difficulties parties face in adopting democratic innovations, particularly when managing the pragmatic concerns of electoral politics (Junius, Caluwaerts, & Erzeel, 2024). ^23^ Additional challenges include the risk of instrumentalizing deliberative tools to bypass traditional barriers to party reform (Rangoni *et al.*, 2024), ^24^ internal polarization (Pârvu & Miscoiu, 2024), ^25^ organizational underdevelopment (Kukec & Čakar, 2024), ^26^ and the very “culture of verticality” within the party, together with a lack of familiarity with forms of deliberative participation (Nasi, 2026). ^27^

Such a high level of demand may be sustainable for militants, but it probably lacks the capacity to include different and lighter forms of supporting a political party. As Scarrow suggests in explaining her “multi-speed membership” model:

“The new affiliation categories introduced […] differ from traditional memberships in that their costs are low […] and do not require long-term commitments. Instead, they offer immediate opportunities for participation and communication.” (Scarrow, 2015: 136) ^10^

Beyond full party members, Scarrow (2015) ^10^ identifies “light members”, characterized by reduced financial contributions and correspondingly reduced participatory rights. “Cyber-members” are recruited and remain active predominantly through online portals and virtual branches, their engagement mediated almost entirely by digital infrastructure. “Sustainers” maintain a relationship with the party that is primarily financial, contributing through one-time or recurring donations without necessarily participating in broader organizational life. Finally, “followers” and “news audiences” represent a more passive category of supporters who register to receive top-down communications via social media platforms, blogs, and newsletters, engaging with the party as consumers of information rather than as active participants.

One of the key challenges with deliberation is that it represents a highly demanding form of participation. Deliberative processes (such as citizens’ assemblies) require financial and temporal resources that political parties often lack at the national or local level, like skilled facilitators and extended timeframes. Parties are frequently required to respond swiftly to emerging events. The mediatization of politics (Mazzoleni & Schulz, 1999) ^28^ imposes timely reactions; otherwise, media and other actors sideline the party. Deliberative processes around timely issues would result in delays incompatible with contemporary political communication’s fast-paced logic.

Another issue often overlooked by proponents of a deliberative model within political parties is maintaining a unified political line. Citizens identify a party based on its stance and the “ownership” of key policy issues (Petrocik, 1996), ^29^ but what happens if one local branch, through a deliberative process, adopts a position that directly contradicts another branch? What if deliberation leads the national party to take a stance at odds with its regional branches or with its allies at the European level? In some cases, extensive internal deliberation may water down the party’s identity, leading to inconsistency and ideological instability, undermining its credibility and coherence in front of the public. Parties must preserve a certain degree of coherence in values and policy proposals to communicate effectively with the electorate and position themselves as credible advocates of specific issues, maintaining the identity of their franchise (Carty, 2004). ^30^

Problems have also been observed with the digitalization of political parties. Those parties that have adopted comprehensive platforms with a range of functionalities have often fallen short of their intended outcomes (Deseriis, 2020). ^31^

According to Gerbaudo, online democracy risks degenerating into a “reactive democracy” (Gerbaudo, 2022) ^32^ or “plebiscitarianism 2.0”, where member involvement is primarily passive or reactive, limited to ratifying decisions made by the leadership (2019) ^33^ (2021a). ^34^ The leader (or “hyperleader”) maintains tight control over the management of the online decision-making process, able to influence outcomes through the manipulation of timing, the tendentious framing of questions, and the capacity to select and modify proposals from the rank-and-file, with generally supermajority results in favor of the leadership’s position. This discrepancy between the idealistic discourse of participatory democracy and the reality of centralized management fuels disillusionment among members and a consequent significant decline in participation in consultations.

We see clear examples of these tendencies in two of the main digital parties that emerged in the last decade in Western Europe: Podemos (Spain) and the Five Star Movement (Italy). Podemos has undergone a deep crisis. Its digital platforms (such as Plaza Podemos) have seen a sharp decline in activity (Meloni & Lupato, 2023). ^35^ Internal voting has also suffered from steadily decreasing participation rates, indicating a growing disengagement among the party’s base (Caiani *et al.*, 2022). ^36^ A similar trajectory can be observed in the case of the Five Star Movement. Although it remains a relevant actor in the Italian political landscape, little remains of the direct participatory vision originally promoted by its founders. Over time, the party has increasingly consolidated around the charismatic leadership of former Prime Minister Giuseppe Conte, making it more and more similar to a personal party (Calise, 2015) ^37^ (Gerbaudo, 2021b). ^38^

What deliberative innovations and comprehensive digital platforms share is a tendency to set expectations too high. They create tools that are often too demanding for the structural and hierarchical (or, at least, stratarchical) limitations of political parties (Carty, 2004) ^30^ (Bolleyer, 2012). ^39^ This hierarchy is necessary for maintaining a unified policy line and cohesion, a dynamic the mere introduction of a technological tool cannot alter. This is not to diminish the value of deliberative processes or comprehensive digital platforms within parties. Digital crowdsourcing here is not proposed as an absolute alternative, but as a complementary strategy. It is important to recognize the practical limitations of every democratic innovation, with the goal of expanding the range of tools available in the repertoire of every party interested into promoting innovative practices.

## Digital crowdsourcing: what are we talking about?

The definition of crowdsourcing comes from an article published on *Wired*, describing it as an open call through which organizations outsource functions previously performed internally to a distributed online crowd (Howe, 2006). ^40^ Aitamurto (2012) ^41^ emphasizes the essential features of crowdsourcing in democratic contexts: the process must be conducted online, participation must be open to all, and the crowd should not be a pre-selected or closed group, but rather a self-selecting, potentially diverse public. This definition aligns with Brabham’s (2008) ^42^ framing of crowdsourcing as a distributed problem-solving and knowledge-production model, also echoing the idea of “wiki-government” (Noveck, 2009). ^43^ Further refinement of this concept includes the idea of “crowdsourced deliberation” (Aitamurto & Landemore, 2016), ^44^ a form of online, asynchronous engagement among self-selected participants aimed at influencing laws and policies in a more reasoned way, and the “CrowdLaw” framework (Alsina & Martí, 2018), ^45^ which emphasizes the use of digital platforms to incorporate citizen input at all stages of the policymaking cycle, from agenda setting to implementation.

It is useful to distinguish between digital crowdsourcing and comprehensive digital platforms. Digital crowdsourcing operates as a focused methodology for co-creation, typically deployed for a specific purpose. It is characterized by its open-ended nature, inviting participants to contribute with ideas and collaboratively build solutions. In contrast, digital platforms like the Five Star Movement’s *Rousseau* or Podemos’ *Plaza Podemos*, represent all-in-one digital ecosystems with various functions (including forums, personal pages, internal referenda, crowdsourcing tools, etc.) within a single, party-controlled architecture. The critical difference lies in the systematic nature of comprehensive platforms. A crowdsourcing process can be arbitrarily activated around a specific issue, whereas digital platforms concern the entire life of the party. Although this may initially appear beneficial in providing more digital democratic affordances (Deseriis, 2021) ^46^ in practice it risks becoming counterproductive, triggering the very mechanisms discussed in the previous section: reactive democracy, plebiscitarianism, hyper-leaderism and excessive expectations.

Digital crowdsourcing is a more flexible and potentially more effective tool. Its project-based nature allows a party to target engagement precisely where it is most needed, without the organizational demands or political risks associated with a full-scale platform reform. Since a crowdsourcing initiative is temporally and topically bounded, it poses less of a threat to the party’s necessary hierarchical cohesion, mitigating the internal resistance that may derail more ambitious digital democratization projects.

Existing works highlight the potential value of crowdsourcing along multiple dimensions. According to Aitamurto and Chen (2017), ^47^ crowdsourcing in policymaking generates democratic value by enhancing transparency, inclusiveness, and accountability; epistemic value by serving as a mechanism for knowledge discovery and policy learning, and economic value by improving efficiency in policy development. Scholars studying the Senador Virtual platform in Chile found that crowdsourcing can help gather diverse perspectives, stimulate informed public debate, and serve as a communicative bridge between citizens and legislators. These processes, when well-designed, can complement formal institutions and provide alternative channels for political expression, especially when traditional participatory mechanisms are weak or exclusionary (Feddersen & Santana, 2022). ^48^

Recently, new opportunities offered by artificial intelligence emerged in enhancing crowdsourcing processes, specifically through integrating human and machine-generated feedback. Bjarnason *et al.* (2024) ^49^ refer to this as *smarter crowdsourcing*, a form of crowdsourcing that leverages computational power and AI-driven insights to reduce the time and cost associated with problem definition, expert engagement, and the synthesis of public input. Other opportunities include the increasing capacity of large language models (LLMs) to summarize extensive discussions, identifying themes, and producing reports that preserve nuance and context (Turan & McKenzie, 2024). ^50^

However, some limitations often mark the implementation of digital crowdsourcing. One of the central concerns is the extent to which these platforms are genuinely participatory or whether they reproduce existing power asymmetries. Asmolov (2015) ^51^ introduced the concept of *vertical crowdsourcing* to describe top-down approaches in which government or institutional actors define the goals, structure, and boundaries of participation, thereby limiting user agency and embedding forms of passive engagement into platform design.

Crowdsourced participation may be merely symbolic, especially when there are no formal mechanisms for integrating public input into decision-making processes (Arnstein, 1969). ^52^ Some studies stress the lack of institutional integration and continuity in crowdsourcing projects. Efforts that are not embedded within formal policymaking channels often result in pseudo-participation (Palacin *et al.*, 2020), ^53^ where citizen input is collected but rarely used in a meaningful way (Christensen *et al.*, 2015) ^54^ (Aitamurto *et al.*, 2017). ^55^ Even successful instances of public engagement, such as the Icelandic or Chilean constitutional processes, have been undermined by political resistance or institutional inertia.

Another recurring issue is the problem of representativeness (Albrecht, 2006). ^56^ Participants in digital crowdsourcing initiatives tend to be disproportionately educated, professionally employed males interested in the policy area considered, leading to the risk of marginalizing low-income and ethnically diverse communities (Pak, Chuca & Moere, 2017). ^57^ More recent research, however, complicates this picture. In the context of online petitioning, women emerge as more active both as initiators and signatories than men, and crowdsourcing participants tend to skew younger. This user base is also comparatively more diverse than that associated with traditional institutional politics (Vromen, Halpin & Vaughan, 2022). ^58^ As Vromen and colleagues summarize: “[…] these actions are still largely driven by the highly educated but are more representative of women’s and younger people’s engagement, as well as being more culturally diverse” (Vromen, Halpin & Vaughan, 2022: p. 46). ^58^ In any case, this point raises questions about whose voices are amplified through these platforms and whether they genuinely reflect the broader public. Demographic skews raise questions about whose voices are amplified through these platforms and whether they genuinely reflect the broader public. Without explicit efforts to broaden access and mitigate digital divides, crowdsourcing may reinforce rather than reduce participatory inequalities.

## Digital crowdsourcing in public institutions and political parties

When dealing with digital crowdsourcing, scholars mainly focused on government-led initiatives, with no attention devoted to political parties as potential users of these tools. The Icelandic constitutional process is one of the most frequently cited examples. Public contributions via social media and online platforms influenced nearly 10 percent of the final constitutional draft, especially on rights-related issues (Morris, 2012) ^59^ (Hudson, 2018). ^60^ Despite the significant engagement, the draft constitution was ultimately not ratified, revealing the fragility of participatory innovations when they lack formal political backing. In Chile, the TuConstitución platform succeeded in mobilizing substantial citizen participation and generated a wide array of public proposals. Once again, despite high levels of engagement, the initiative ultimately failed to secure sustained institutional uptake or exert long-term influence on constitutional reform processes (Pogrebinschi, 2023). ^61^

Another frequently cited case is the use of the Pol.is machine learning system in Taiwan through the vTaiwan project. This platform has been employed to identify consensus areas among diverse citizen groups and to provide structured input for policymaking on salient issues, including the regulation of ride-sharing services such as Uber and the formulation of ethical principles for artificial intelligence (Moats & Tseng, 2023). ^62^

Little attention has been devoted to examining how crowdsourcing might be used internally by political parties. Pirate parties have been among the most active adopters of crowdsourcing, experimenting with a range of participatory mechanisms. Examples include the Policy Beta platform, developed in the United Kingdom to crowdsource policy proposals, and the “swarm intelligence” model advanced by the Swedish Pirate Party, which draws on crowdsourcing across a variety of functions (including fundraising, content creation, and organizational scaling (Falkvinge, 2013). ^63^ Even though it was not formally a party, in 2018 the left-wing political organization Aufstehen utilized Pol.is to gather citizens’ opinions for informing its political agenda. Aufstehen (“Stand Up”) was a left-wing political organization launched in Germany in 2018 by Sahra Wagenknecht after her departure from the Left Party (Die Linke). Following that, in 2023, Wagenknecht established Bündnis Sahra Wagenknecht (BSW) first as a political association, and then as a political party (Thomeczek, 2024). ^64^ In 2019, the Austrian People’s Party (ÖVP) launched *Evolution Volkspartei*, an initiative using the digital platform Ideenwand for the co-creation of the new party program, allowing citizens to register, submit ideas, comment on, and evaluate others’ contribution (Schmidthuber *et al.*, 2018). ^65^ More recently, in 2024, the pan-European party Volt introduced EuroSense, a narrative sense-making tool designed to capture the concerns, passions, and experiences of individuals across Europe.

Crowdsourcing within public institutions and political parties exhibits both similarities and differences. They share fundamental mechanisms such as the potential to influence public policy and the need to adopt such processes to strengthen connections with citizens and rebuild relationships of trust. However, crowdsourcing may work even more effectively for political parties, given that, unlike institutions such as parliaments and governments, they are private organizations and therefore enjoy greater flexibility, both organizationally and in terms of the issues they can address. On the other hand, parties possess a lower degree of legitimacy compared to public institutions (OECD, 2024). ^66^ This may make it more difficult to achieve broad outreach and genuine engagement, particularly among individuals who feel distant from the party. Another distinction between parties and public institutions concerns the relationship between participatory processes and political decision-making. In the case of a government, a participatory process that produces a proposal can more directly led to specific policy to be implemented. By contrast, parties face an additional layer. When a party carries out a participatory process, the resulting proposal cannot be implemented right away: it first needs to gain governmental power before it can turn that proposal into action.

## A typology of digital crowdsourcing for political parties

To better understand the role of digital crowdsourcing within political parties, we can conceptualize a typology (Collier *et al.*, 2012) ^67^ based on two key dimensions: policy impact and power structure.

The first dimension is policy impact, namely the influence of the crowdsourcing initiative on concrete political decisions, such as policy formulation or a party’s electoral program. For a participatory process to be considered genuinely effective, it must demonstrate a clear pathway from public input to political output. The necessity of impact is particularly acute for crowdsourcing. While in other participatory models, such as deliberative forums, the value may also lie in the process itself (e.g., fostering dialogue, civic education, and community building), crowdsourcing is a more results-oriented tool. We can therefore distinguish between two outcomes. *Low policy impact*, when the process yields no substantive effect on final decisions, and *high policy impact*, when the crowdsourced input directly informs and leads to concrete political actions.

The second category pertains to the organizational structure of the process, specifically, the extent to which it gathers citizens’ input in a bottom-up manner, as opposed to subtly guiding the conversation toward predefined goals. This dimension allows us to assess the democratic character of a crowdsourcing process—specifically, the extent to which it redistributes power to a broader base of participants. Taking into account power structure implies looking at the structural features of the process rather than its stated intentions. It also requires consideration of the initiative’s actual capacity to engage a broad and diverse community, and not just those already accustomed to participating in party politics (who often possess a greater amount time, money, or civic skills (Brady *et al.*, 1995). ^68^

We can identify two ideal-typical models. In a *top-down power structure*, the process is organized to ensure that outcomes align with pre-defined expectations. In this model, the results are engineered to reinforce established power relations and cannot fundamentally challenge the organizing authority. This can be achieved through a stringent agenda-setting, where the institution defines the specific, narrow question to be answered, leaving no room for participants to redefine the problem, or by including just a narrow community of already-aligned people. Another method is curatorial control, where the institution filters, synthesizes, or modifies the crowdsourced proposals before they are finalized, or uses a ratification-style vote that presents a single, pre-packaged proposal for a simple yes/no decision with a clear preferred answer by the party elite. Conversely, in a *bottom-up power structure* the process is designed to prevent the organizing elites from determining the outcomes. This structure creates a genuine opening for the participant base to define problems, generate ideas, and exert a tangible influence on the final results. This is typically enabled by an open-ended agenda, allowing the community to identify and prioritize the issues for discussion. The process may also incorporate community-based moderation and filtering, where participants themselves can up-vote, comment on, and refine proposals, determining which ideas rise to the top.

**Figure 1.**
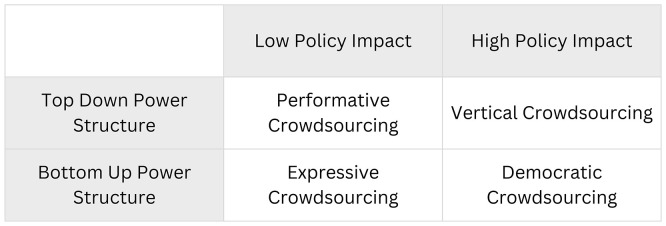
A typology of digital crowdsourcing for political parties.

The combination of these two dimensions gives rise to four types of digital crowdsourcing for political parties, as shown in Figure 1: vertical crowdsourcing, performative crowdsourcing, expressive crowdsourcing, and democratic crowdsourcing.

When a digital crowdsourcing initiative demonstrates a tangible impact on policy or party direction yet operates under a top-down power structure, it can be classified as *vertical crowdsourcing.* This term, introduced by Asmolov (2015), ^51^ describes a model where the efficiency of outcomes is achieved at the expense of participants’ autonomy. In this framework, the process is designed to channel participation toward a pre-approved outcome, ensuring that the results are aligned with the leadership’s existing agenda. A typical example is a consultation hosted on a political party’s digital platform where the central question is narrowly defined by the leadership and framed in a way that predisposes participants to a specific conclusion. For instance, rather than asking an open-ended question like “How should we address climate change?”, the prompt might be “Do you support our party’s proposed Green New Deal to create jobs and save the planet?”

When a digital crowdsourcing process is characterized by a top-down power structure and also yields minimal influence on policy (low policy impact), we see an example of *performative crowdsourcing.* Its function is strategic communication, allowing a political organization to project an image of openness without ceding any actual decision-making authority. Participation is performed as a communication strategy, rather than being a concrete mechanism for influence. For instance, a party may launch a consultation that mirrors the controlled structure of vertical crowdsourcing with a predetermined agenda and tightly framed questions, but designates the results as non-binding. In another case, a party might publicly announce a participatory initiative with great fanfare, only to invest insufficient resources, provide limited publicity, or fail to integrate the results into any subsequent decision-making process, effectively abandoning it after the initial public relations benefit has been reaped.

The third type, *expressive crowdsourcing*, emerges when a process features bottom-up power dynamics but fails to achieve significant policy impact. In this scenario, the will to decentralize power and gather authentic citizen input is genuine; however, the initiative lacks the institutional mechanisms or political will to translate this input into binding decisions. This disconnect may stem from the party’s structural incapacity to integrate public feedback or from a deliberate choice to keep the process strictly advisory. The value of such initiatives thus becomes expressive, serving as a channel for expressing voices and opinions rather than a direct driver of policy. While this can foster a sense of community and open dialogue, the absence of tangible outcomes carries a significant risk. As scholars like Baiocchi & Ganuza (2014) ^69^ argue, for participation to be truly meaningful and empowering, it must lead to visible impacts.

The final model, *democratic crowdsourcing*, is characterized by the combination of bottom-up power dynamics and high policy impact. In this case, the process is structured to grant participants substantive agenda-setting and decision-making power, with a committed pathway for their input to be translated into formal policy or party directives. Another important element is the inclusion of external individuals, expanding the process to a larger number of people than those who typically take decisions in the party. This integration of inclusive participation with binding outcomes should enhance internal party democracy, potentially strengthening legitimacy and engagement among members and supporters. In the final section of this paper, I address some of the indispensable conditions to achieve this type of crowdsourcing.

## Opportunities and challenges of digital crowdsourcing

Having established a typology of digital crowdsourcing, this section examines its distinct opportunities as a tool for political parties. The analysis will focus on three advantages: new venues for members and non-members to contribute to the party positions; increased flexibility, which allows parties to tailor initiatives to specific contexts and objectives; and accessibility, which lowers barriers to entry.

### Opportunities

The first benefit of digital crowdsourcing is more room for bottom-up *democratic participation.* Digital crowdsourcing provides a structured mechanism for systematically gathering the opinions, voices, and contributions of citizens and members. Its open-ended nature is particularly suited to collecting nuanced and complex inputs. In this way, political parties gain a deeper, more qualitative understanding of their supporters’ concerns, while citizens acquire a tangible channel to express their views and potentially shape political agendas. Compared to comprehensive digital platforms, a discrete crowdsourcing initiative presents distinct strategic advantages. Its focused nature makes it easier to manage, communicate, and promote to a broad public. With sufficient political and financial investment, such a targeted initiative can potentially reach a wider audience than a more diffuse platform. A successful crowdsourcing campaign can demonstrate that the party is not a closed or entrenched entity, but one actively seeking external input, generating positive feedback loops of participation.

The second opportunity is *flexibility.* Digital crowdsourcing offers a degree of flexibility that distinguishes it from more structured participatory instruments, such as deliberative assemblies or digital platforms. When thoughtfully designed, it can be adapted to a wide spectrum of political objectives. A party might deploy it to refine a policy position, solicit input on internal party reform, gather design-oriented feedback on its visual identity, establish criteria for candidate selection, or brainstorm the implementation and communication of legislation. It can also serve to gauge public sentiment on specific issues or collect personal narratives and stories and can be used to inform the development of a party’s overarching narrative and identity. Moreover, digital crowdsourcing processes can operate in parallel with in-person initiatives, thereby facilitating the inclusion of individuals who are less tech savvy.

This adaptability is important for political parties. Unlike comprehensive digital platforms that establish a permanent structure for participation (which can become politically challenging to honor when a party enters government) crowdsourcing offers a more modular approach. It allows party elites to calibrate participation in a way that is coherent with their political constraints, deploying it for less sensitive topics or for feedback collection without the high-stakes commitment of a binding comprehensive digital platform. Obviously, crowdsourcing is less suited for fostering the deep, discursive deliberation and negotiation required for complex, value-laden issues. In such contexts, deliberative democratic innovations remain the more appropriate choice.

The third opportunity of this technology is *accessibility.* Another strength of digital crowdsourcing is its low barrier to entry, opening the doors to less-engaged party supporters (Scarrow, 2015). ^10^ When the process is designed for simplicity (requiring only a brief, user-friendly online contribution without complex registration) it significantly broadens accessibility and enhances participation rates. This agility makes it fundamentally more inclusive than demanding models like deliberative mini-publics or formal consultations, which require substantial time, training, or formal membership. Deliberative formats, particularly sortition-based mini-publics, are inherently limited in size, involving only a small, though statistically representative, group of participants (Escobar & Elstub, 2017). ^70^ Similarly, digital party platforms have traditionally been designed for and used by existing party members. In contrast, a well-publicized and credible crowdsourcing initiative can engage a much wider external audience, attracting contributions from citizens who would not otherwise participate in formal party structures but are motivated by an opportunity to have their voice heard. It is important to note, however, that accessibility alone is insufficient. While lowering the cost of participation facilitates broader engagement, it is not a panacea for political disconnection. As Tufekci (2017) ^71^ argued, such “light-touch” digital participation can reduce the opportunity to build the strong social bonds and collective identity that underpin sustained collective action.

### Challenges

This sub section examines three specific challenges of digital crowdsourcing for political parties: the inclusion-exclusion dilemma arising from opening party processes to non-members; the persistent risk of elite capture inherent in the tool’s nature; and different views on democratic legitimacy, which highlights the conflict between bottom-up inputs and a party’s need for hierarchical representation.

The first challenge in implementing crowdsourcing within political parties lies in managing the tension between the inclusive potential of such tools and the inherent exclusivity of political parties. I refer to this problem as the *inclusion-exclusion tension.* By definition, a party is an exclusive entity (Rosenblum, 2009). It represents a specific segment of the electorate and society, and this exclusivity plays a key role in fostering a sense of belonging among its members, providing them with incentives and avenues for political engagement (Weber, 2020). ^72^ Therefore, when participation is opened to everyone, issues of democratic legitimacy and party ownership arise. For instance, a party member may be legitimately disappointed with a non-member having the same power and voice in a participatory process. This tension is exacerbated by the fact that open participation increases the risk of manipulation, particularly through mass engagement by individuals or groups seeking to undermine or hack the decision-making process. Malicious actors may be even more prevalent in participatory initiatives launched by a political party than in those initiated by public institutions, since the pool of individuals with negative sentiments or attitudes toward a specific party is likely to be larger than the pool of those opposed to public institutions.

In addition to the risk of manipulation from below, a second concern in digital crowdsourcing is the potential for *elite capture*, or *manipulation from above.* As a participatory tool, digital crowdsourcing is highly susceptible to elite influence, particularly through selecting topics, framing questions, and structuring the process. In addition, parties may use crowdsourcing to collect and organize participants’ contact information, which can then serve as a resource for communication about party activities, electoral campaigns, and internal organizational matters, as well as for targeting potential voters. While this practice may carry democratic potential (for example, by enabling parties to better understand and engage their support base) it also entails the risk that such data may be used in opaque or non-transparent ways. Democratic crowdsourcing can therefore easily translate into vertical or performative crowdsourcing. This shift may occur not out of deliberate intent, but rather due to limited awareness of the instrument’s sensitivity to power imbalances: for example, insufficient investment to ensure meaningful inclusion of typically excluded groups, or the absence of a clearly defined and publicly communicated mechanism for how outputs will be used. Transparency then is a key factor to prevent elite capture, so that outputs are not arbitrarily changed according to specific desiderata.

The third challenge digital crowdsourcing faces relates with *different views on democratic legitimacy.* Political parties face the imperative of maintaining coherent policy positions. From this perspective, digital crowdsourcing can become a potentially problematic tool, especially when the feedback collected contradicts the party’s established positions. For example, if a progressive party has made the defense of migrant rights a central pillar of its platform, but the feedback from citizens reveals widespread hostility toward migrants, is the party then expected to change its stance?

The tension here is not simply one between “more democracy” and “less democracy” but rather between different forms of democratic legitimacy. The consistency of the party line (and the avoidance of continuous contestation of key decisions through participatory mechanisms) is a prerequisite for collective action rooted in the principle of representation. Historically, this principle has always implied a degree of autonomy for elected officials to prevent a descent into the so-called “tyranny of the majority” (Manin, 1997). ^73^ Here we see a clash between two democratic ideals. On one side is representative democracy, in which a party’s internal democracy culminates in the selection of a national leader who then enjoys substantial autonomy in setting and pursuing policy priorities. On the other side is participatory (or even direct) democracy, which emphasizes the role of ordinary citizens in continuously shaping a party’s policy positions. The key question is whether these two models can be reconciled within the structure of a political party. In the next and final section of this paper, I will suggest strategies for making this conciliation possible.

## Towards a feasible democratic crowdsourcing for political parties

To make the most of its strengths, parties can adopt specific strategies that move toward a more democratic form of crowdsourcing, one that gathers input from citizens and has real political impact while respecting the party’s identity, values, and internal organization.

*The first strategy is to support gradual implementation and to focus on specific topics.* The main advantage of digital crowdsourcing over comprehensive digital platforms is that it does not require a party to open its entire agenda to participatory input. Instead, parties can strategically focus consultations on specific issues where there is a genuine willingness to incorporate external contributions. Attempting to crowdsource an entire party program often proves unfeasible and results in unmet expectations. A more effective strategy is to proceed incrementally, beginning with less contentious, broadly supported topics, such as a social democratic party initiating a consultation on raising the minimum wage before addressing more polarizing issues like immigration. This is the case for valence issues, namely those topics on which there is broad cross-cutting agreement about the desired objective, but disagreement over which political actor is most competent and trustworthy to achieve it (Stokes, 1963). ^74^ Unlike position issues (where competing policy preferences structure political conflict) valence issues are less likely to produce internal divisions. For this reason, initiating a crowdsourcing process on valence issues may allow a party to meaningfully incorporate citizens’ input while minimizing the risk of undermining its policy stances. Crucially, managing expectations is vital, as overly ambitious promises are likely to be disappointed, thereby undermining both the immediate initiative and the party’s broader credibility for future participatory initiatives.

*The second strategy is to establish a clear and reliable mechanism for translating inputs into tangible outputs.* For crowdsourcing to be genuinely democratic, participants’ inputs need to be translated into political decisions, establishing a high level of policy impact. The credibility and effectiveness (and, consequently, level of participation) of the initiative depend on transparently communicating this linkage from the outset. Prospective participants require a clear understanding of how their contributions will be utilized, as this clarity serves as a fundamental incentive for engagement. Without the realistic prospect of a tangible outcome, participation is likely to be perceived as inconsequential, ultimately discouraging citizen involvement and undermining the process’s validity and credibility.

*The third strategy concerns how to limit participation.* Crowdsourcing implies an open and inclusive form of participation. However, this can sometimes be at odds with specific structural and normative features of political parties. An intermediate solution could, therefore, be considered, one that does not restrict participation exclusively to formal members but allows broader involvement from those willing to endorse a set of core values aligning them with the party’s political positions, such as “sustainers” or “followers” may be (Scarrow, 2015). ^10^ This could be achieved through simple actions, such as ticking a box before accessing the process. Identity verification mechanisms might also be considered to reduce the risk of manipulation from below. Although such measures could compromise the ease and user-friendliness of the platform, they may become necessary at later stages. Initial phases of experimentation could remain open to everyone, and if high levels of participation are observed, some form of identity check could be introduced subsequently to safeguard the process from malicious trolling and algorithmic hacking, which probably represent the greatest threat for the success of these initiatives.

The fourth (and probably most important) strategy is to think of digital crowdsourcing as a political and social process, not as a technical fix. One of the central challenges in employing technology for democratic participation is the assumption that mere adoption is sufficient (Randma-Liiv, 2023). ^75^ This approach risks slipping into a form of “technological fixism”, whereby technical tools alone are presumed capable of resolving complex social problems (Pacey, 1983). ^76^ This assumption is a direct path to failure. Participation is, above all, a social process that must account for elites’ political will, individuals’ attitudes and biases, their incentives to engage, the collective imaginaries surrounding particular issues, and the broader dynamics of public opinion. For example, any digital crowdsourcing initiative must be attuned to ongoing national debates. If public discourse at a given moment is dominated by concerns about national security, launching a participatory process on welfare reform is unlikely to gain traction. This also entails a significant commitment to developing an effective communication strategy. A frequent pitfall of participatory initiatives is the assumption that individuals will spontaneously engage. In practice, participation must be actively nurtured through targeted communication, outreach, and efforts to cultivate a sense of belonging and efficacy among potential contributors. Similarly, to be effective and inclusive, digital crowdsourcing tools must be designed to be as user-friendly as possible; otherwise, they risk remaining elitist and inaccessible to large parts of the population.

## Conclusion

In this paper, I sought to connect research on digital crowdsourcing with the debate on intra-party democracy, deliberative democratic innovations and digital platforms. I argued that digital crowdsourcing may be better suited to advancing internal democracy than deliberative processes or comprehensive digital platforms. However, these instruments should not be viewed in opposition to one another. Depending on its political culture and priorities, each party should determine its own balance among different participatory strategies. Digital crowdsourcing is not a definitive solution but rather one additional tool in a broader repertoire, carrying its own advantages and limitations.

We should always remember how it is inherently challenging for political parties to adopt such tools. This reluctance can be explained not only by internal power dynamics, but also by the very nature of political parties. Parties are conservative organizations (Panebianco, 1988). ^14^ Their hierarchical structure serves the goal of maintaining clear policy positions. Evidence shows that support for democratic innovations is quite minoritarian, and “heavily related to anti-establishment parties, to left-wing parties and to parties with limites access to power” (Núñez, Close & Bedock, 2016, p. 1). ^77^ Furthermore, even when their value and potential are acknowledged, participation and internal democracy rarely become primary goals.

As Harmel and Janda (1994) ^13^ argue, party change depends on parties’ primary goals. In turn, these goals may be shaped by a shift in the dominant coalition within the party or by institutional and organizational factors within the political system. More generally, however, parties’ primary goals tend to be outward-oriented, focused on vote-, office-, or policy-seeking. As a result, parties are often poorly equipped and incentivized to pause, reflect, and undertake substantial internal reforms. How, then, can an interest in a party’s own democratization become a priority? Most likely, to the extent that such a goal can go hand in hand with more immediate or pressing objectives, such as vote-seeking and policy-seeking. The adoption of participatory instruments such as crowdsourcing, therefore, should not be understood solely as a tool for strengthening internal democracy. It may also serve other goals, particularly improving policy development (policy-seeking) and projecting a more favorable image to the electorate (vote-seeking). By contrast, it may be less useful for parties that are primarily oriented toward office-seeking, insofar as it contributes little to their stability as governing partners.

### Ethics and consent

Ethical approval and consent were not required to this paper.

## Data Availability

All data underlying the results presented in this study are contained within the article.
