# Supplementary material for: Less is more. Exploring opportunities and challenges of digital crowdsourcing for political parties
Source: Open Res Eur. 2026 Apr 16;5:397. Originally published 2025 Dec 22. [Version 2] doi: 10.12688/openreseurope.22121.2 (PMC12873537; doi:10.12688/openreseurope.22121.2)
Supplement: Peer review history

# Reviewer report: Reviewer response for version 2

**DOI:** 10.21956/openreseurope.25303.r72466

**Published:** 2026-04-25

**Authors:** Jasmin Fitzpatrick

**Affiliations:** 1 Johannes Gutenberg-Universität Mainz, Mainz, Germany

The author has thoughtfully incorporated all prior feedback: linking to Harmel & Janda (1994) and Scarrow (2015), updating demographics with Vromen et al. (2022), correcting the Aufstehen/BSW distinction, and refining the challenge section with valence/position issue insights. The paper remains well-structured, timely, and theoretically grounded. It makes a valuable contribution to party research by exploring how crowdsourcing can enhance intra- and extra-party democracy. I recommend acceptance without reservation.

If applicable, is the statistical analysis and its interpretation appropriate?

Not applicable

Is the study design appropriate and is the work technically sound?

Yes

Is the work clearly and accurately presented and does it engage with the current literature?

Partly

Are the conclusions drawn adequately supported by the results?

Yes

Are sufficient details of methods and analysis provided to allow replication by others?

Not applicable

Are all the source data and materials underlying the results available?

No source data required

Reviewer Expertise:

Political Party Research, Political Organization, Political Participation, Political Communication

I confirm that I have read this submission and believe that I have an appropriate level of expertise to confirm that it is of an acceptable scientific standard.

# Reviewer report: Reviewer response for version 2

**DOI:** 10.21956/openreseurope.25303.r72467

**Published:** 2026-04-17

**Authors:** Michal Malý

**Affiliations:** 1 Charles University, Prague, Czech Republic

I appreciate that the revised manuscript now incorporates the Pirate Parties and the discussion of swarm logic, which strengthens the illustrative section on digital crowdsourcing in political parties. I also welcome the clearer engagement with the party change literature, particularly through the inclusion of Harmel and Janda, as this helps better situate the argument within broader debates on strategic adaptation and organisational change. Finally, the addition of the instrumental dimension of contact harvesting and its possible strategic uses usefully sharpens the discussion of elite control, co-optation, and the democratic limits of participatory design.

If applicable, is the statistical analysis and its interpretation appropriate?

Not applicable

Is the study design appropriate and is the work technically sound?

Yes

Is the work clearly and accurately presented and does it engage with the current literature?

Yes

Are the conclusions drawn adequately supported by the results?

Yes

Are sufficient details of methods and analysis provided to allow replication by others?

Not applicable

Are all the source data and materials underlying the results available?

No source data required

Reviewer Expertise:

Political Parties, Digital Politics, Synthetic Politics

I confirm that I have read this submission and believe that I have an appropriate level of expertise to confirm that it is of an acceptable scientific standard.

# Reviewer report: Reviewer response for version 1

**DOI:** 10.21956/openreseurope.23943.r68316

**Published:** 2026-02-04

**Authors:** Michal Malý

**Affiliations:** 1 Charles University, Prague, Czech Republic

The manuscript tackles a timely, highly relevant topic and presents its argument clearly, coherently, and in a well-structured manner. I found the conceptual discussion persuasive—especially the positioning of digital crowdsourcing as a comparatively “lightweight” participatory tool when contrasted with more demanding deliberative procedures and all-in-one party platforms, and the proposed typology is analytically useful and easy to operationalise across different empirical contexts.

 I only have 3 short comments to consider:

1. In the section “Digital crowdsourcing in public institutions and political parties,” where the author briefly lists illustrative examples, I would recommend adding Swarmwise (Falkvinge 2013) as an additional reference, because it explicitly frames the organisational logic of early Pirate parties through crowdsourcing/swarm-like participation and therefore fits well alongside the other examples the author provides.
2. While the manuscript notes that parties adopt these tools in pursuit of party goals, I would encourage the author to anchor this point more explicitly in the party change literature, particularly Harmel and Janda (1994), to better specify how strategic incentives, adaptation pressures, and organisational objectives intersect with (and sometimes override) democratic-participatory rationales.
3. I would suggest adding one explicit, instrumentally oriented motivation that is often central in practice but is only implicit here: parties may deploy crowdsourcing not only to generate input or signal openness, but also to harvest and consolidate participant contacts (supporter lists, mailing lists, mobilization channels), which can then be leveraged for continuous communication about party activities, campaigns, and organizational matters; acknowledging this mechanism would (in my opinion) further sharpen the paper’s discussion of strategic uses and potential co-optation of participatory design.

The manuscript is well argued, conceptually clear, and addresses a timely and important topic; once the above points are taken into account, I see no reason not to recommend the article for acceptance.

If applicable, is the statistical analysis and its interpretation appropriate?

Not applicable

Is the study design appropriate and is the work technically sound?

Yes

Is the work clearly and accurately presented and does it engage with the current literature?

Yes

Are the conclusions drawn adequately supported by the results?

Yes

Are sufficient details of methods and analysis provided to allow replication by others?

Not applicable

Are all the source data and materials underlying the results available?

No source data required

Reviewer Expertise:

Political Parties, Digital Politics, Synthetic Politics

I confirm that I have read this submission and believe that I have an appropriate level of expertise to confirm that it is of an acceptable scientific standard, however I have significant reservations, as outlined above.

## References

[1] : Swarmwise: The tactical manual to changing the world. *https://www.researchgate.net/publication/228809041_Prosodic_parameters_for_the_detection_of_regiona…* .

# Response

**Published:** 2026-03-06

**Authors:** Francseco Nasi

**Affiliations:** Political and Social Sciences, Universita degli Studi di Bologna Dipartimento di Scienze Politiche e Sociali, Bologna, Emilia-Romagna, Italy

With regard to Reviewer 3’s suggestions:

 1) I added the Swedish Pirate Party to the list of parties using crowdsourcing strategies, also mentioning the UK Pirate Party and the Policy Beta platform. The text now reads as follows: Little attention has been devoted to examining how crowdsourcing might be used internally by political parties. Pirate parties have been among the most active adopters of crowdsourcing, experimenting with a range of participatory mechanisms. Examples include the Policy Beta platform, developed in the United Kingdom to crowdsource policy proposals ^i^, and the "swarm intelligence" model advanced by the Swedish Pirate Party, which draws on crowdsourcing across a variety of functions (including fundraising, content creation, and organizational scaling (Falkvinge, 2013).

 2) Concerning the second point, I have integrated works on party change—specifically those by Harmel and Janda—into different parts of the paper, also following Reviewer 2’s suggestion (see point 1). In particular, the conclusion now includes a reflection on how external shocks and primary goals may intersect in promoting change oriented toward democratic rationales and digital crowdsourcing.

 3) Finally, I have incorporated the reviewer’s suggestion in the section discussing the risk of top-down manipulation. The text now reads as follows: In addition to the risk of manipulation from below, a second concern in digital crowdsourcing is the potential for *elite capture*, or *manipulation from above*. As a participatory tool, digital crowdsourcing is highly susceptible to elite influence, particularly through selecting topics, framing questions, and structuring the process. In addition, parties may use crowdsourcing to collect and organize participants’ contact information, which can then serve as a resource for communication about party activities, electoral campaigns, and internal organizational matters, as well as for targeting potential voters. While this practice may carry democratic potential (for example, by enabling parties to better understand and engage their support base) it also entails the risk that such data may be used in opaque or non-transparent ways. Democratic crowdsourcing can therefore easily translate into vertical or performative crowdsourcing

# Reviewer report: Reviewer response for version 1

**DOI:** 10.21956/openreseurope.23943.r66994

**Published:** 2026-01-22

**Authors:** Jasmin Fitzpatrick

**Affiliations:** 1 Johannes Gutenberg-Universität Mainz, Mainz, Germany

The paper tackles a compelling and timely topic by focussing on a theoretic specification of how parties may employ crowdsourcing to enhance intra-party but also beyond-party democracy. The paper is well structured and well informed.

 My understanding is that the paper aims to target a more general audience in party research and therefore I suggest a connection with Harmel and Janda (1994) in terms of their party goal of democracy maximation and the multispeed-membership model by Scarrow (2015). This would enable the scholarly community to learn more about the specific membership types that might contribute to party activities via crowdsourcing.

 Regarding the demographic nature of participants of crowdsourcing, the paper refers to Albrecht (2006). A lot has happened ever since, especially the founding of the parties mentioned in the paper (Podemos, M5S) but also the advancements in the digital sphere have evolved immensely. Therefore, I suggest including the work of Vromen and colleagues (2022) which provides more recent information.

 The paper mentions first efforts by political parties to include crowdsourcing and refers to the German movement *Aufstehen*. *Aufstehen* is a political organization, not a party according to the German Party Law §2. Therefore, this statement requires revision for accuracy. *Aufstehen* marked Sahra Wagenknecht’s political break from Die Linke (The Left Party). It was subsequently succeeded by the founding of BSW (Bündnis Sahra Wagenknecht), which began as a political association before being formally registered as a political party and later contesting elections (sources below).

 The paper points to different chances and challenges of crowdsourcing for parties. Reading the challenge section, I found myself reflecting on whether crowdsourcing might be more effectively applied to certain types of issues — perhaps more suited to valence issues than position issues. This consideration seems particularly relevant in the context of the example discussed in the paper (migration policy). I would therefore gently suggest integrating insights from this broader literature into the paper’s argument, which could help offer a more nuanced and comprehensive perspective on the topic.

 Overall, the paper is very inspiring. Despite the few requests that in my opinion would strengthen the relevance of this article in different areas of party research, I find no compelling reason to recommend against publication — the article is well-argued, timely, and worthy of consideration.

 Weisskircher, Manès. 2018. Will Germans rise up for a new left-wing movement? What to know about Aufstehen. EUROPP. https://blogs.lse.ac.uk/europpblog/2018/08/30/will-germans-rise-up-for-a-new-left-wing-movement-what-to-know-about-aufstehen/

If applicable, is the statistical analysis and its interpretation appropriate?

Not applicable

Is the study design appropriate and is the work technically sound?

Yes

Is the work clearly and accurately presented and does it engage with the current literature?

Partly

Are the conclusions drawn adequately supported by the results?

Yes

Are sufficient details of methods and analysis provided to allow replication by others?

Not applicable

Are all the source data and materials underlying the results available?

No source data required

Reviewer Expertise:

Political Party Research, Political Organization, Political Participation, Political Communication

I confirm that I have read this submission and believe that I have an appropriate level of expertise to confirm that it is of an acceptable scientific standard, however I have significant reservations, as outlined above.

## References

[1] : Bündnis Sahra Wagenknecht (BSW): Left-Wing Authoritarian—and Populist? An Empirical Analysis. *Politische Vierteljahresschrift* .2024;65(3) : 10.1007/s11615-024-00544-z 535-552 10.1007/s11615-024-00544-z

# Response

**Published:** 2026-03-06

**Authors:** Francseco Nasi

**Affiliations:** Political and Social Sciences, Universita degli Studi di Bologna Dipartimento di Scienze Politiche e Sociali, Bologna, Emilia-Romagna, Italy

I have attempted to incorporate all of the second reviewer’s suggestions.

 1) First, with regard to the connection with Harmel and Janda, this point is now addressed both at the beginning and at the end of the paper, thereby situating this work more clearly within that strand of the literature. For example, the second section of the paper now begins as follows: To better situate the potentiality of digital crowdsourcing for political parties, we firstly need to look at the debate on party change and party democratization. As Harmel and Janda (1994) argued, party change largely rests on party goals. Building on Panebianco (1988), they suggest that change (given the conservative natures of parties themselves) generally depends on external shocks, and these shocks are dependent on party primary goals. For vote maximizers, such shocks consist in electoral defeat or a substantial loss of popular support. For office maximizers, they are events “directly related to participation in government” (Harmel & Janda, 1994, p. 270). For policy-seeking parties, the most consequential shocks are those that shake the party’s ideological and identity foundations (as was the fall of the Berlin Wall and the end of Soviet communism for Western communist parties). Finally, for intraparty democracy maximizers, the most significant shocks concern changes in party membership—whether in size or in its social composition—as well as broader societal transformations that “change expectations or resources with regard to participation” (Harmel & Janda, 1994, p. 271), such as the growing demand for new forms of engagement in a digitalized environment may be. The conclusion then revisits their arguments within a broader reflection on how, and to what extent, parties may adopt changes in the direction of digital crowdsourcing: As Harmel and Janda (1994) argue, party change depends on parties’ primary goals. In turn, these goals may be shaped by a shift in the dominant coalition within the party or by institutional and organizational factors within the political system. More generally, however, parties’ primary goals tend to be outward-oriented, focused on vote-, office-, or policy-seeking. As a result, parties are often poorly equipped and incentivized to pause, reflect, and undertake substantial internal reforms. How, then, can an interest in a party’s own democratization become a priority? Most likely, to the extent that such a goal can go hand in hand with more immediate or pressing objectives, such as vote-seeking and policy-seeking. The adoption of participatory instruments such as crowdsourcing, therefore, should not be understood solely as a tool for strengthening internal democracy. It may also serve other goals, particularly improving policy development (policy-seeking) and projecting a more favorable image to the electorate (vote-seeking). By contrast, it may be less useful for parties that are primarily oriented toward office-seeking, insofar as it contributes little to their stability as governing partners.

 2) With regard to the second point, namely the integration of the “multispeed membership model” proposed by Scarrow (2015), I have introduced this perspective in several parts of the paper and incorporated it into the overall argument. From the introduction: Might there be space for forms of political participation that are lighter, more adaptable to the needs of political parties and able to take into account increasingly diverse forms of memberships (Scarrow, 2015)? In section two: Such a high level of demand may be sustainable for militants, but it probably lacks the capacity to include different and lighter forms of supporting a political party. As Scarrow suggests in explaining her “multi-speed membership” model: “The new affiliation categories introduced […] differ from traditional memberships in that their costs are low […] and do not require long-term commitments. Instead, they offer immediate opportunities for participation and communication.” (Scarrow, 2015: 136) Beyond full party members, Scarrow (2015) identifies “light members”, characterized by reduced financial contributions and correspondingly reduced participatory rights. “Cyber-members” are recruited and remain active predominantly through online portals and virtual branches, their engagement mediated almost entirely by digital infrastructure. “Sustainers” maintain a relationship with the party that is primarily financial, contributing through one-time or recurring donations without necessarily participating in broader organizational life. Finally, “followers” and “news audiences” represent a more passive category of supporters who register to receive top-down communications via social media platforms, blogs, and newsletters, engaging with the party as consumers of information rather than as active participants. And finally in different parts of the paper: Another strength of digital crowdsourcing is its low barrier to entry, opening the doors to less-engaged party supporters (Scarrow, 2015) […] endorse a set of core values aligning them with the party's political positions, such as “sustainers” or “followers” may be (Scarrow, 2015)”

 3) As a third point, I updated the section of the paper on the potential risks of digital crowdsourcing by integrating Vromen and colleagues’ book. This is the whole updated part: Participants in digital crowdsourcing initiatives tend to be disproportionately educated, professionally employed males interested in the policy area considered, leading to the risk of marginalizing low-income and ethnically diverse communities (Pak, Chuca & Moere, 2017). More recent research, however, complicates this picture. In the context of online petitioning, women emerge as more active both as initiators and signatories than men, and crowdsourcing participants tend to skew younger. This user base is also comparatively more diverse than that associated with traditional institutional politics (Vromen, Halpin & Vaughan, 2022). As Vromen and colleagues summarize: “[…] these actions are still largely driven by the highly educated but are more representative of women’s and younger people’s engagement, as well as being more culturally diverse” (Vromen, Halpin & Vaughan, 2022: p. 46). In any case, this point raises questions about whose voices are amplified through these platforms and whether they genuinely reflect the broader public. Demographic skews raise questions about whose voices are amplified through these platforms and whether they genuinely reflect the broader public. Without explicit efforts to broaden access and mitigate digital divides, crowdsourcing may reinforce rather than reduce participatory inequalities.

 4) As a fourth point, I corrected the mistake on the political organization Aufstehen. Now the text goes like this (with a note): Even though it was not formally a party, in 2018 the left-wing political organization *Aufstehen ^ii^* (Thomeczek, 2024) utilized Pol.is to gather citizens’ opinions for informing its political agenda ^ii^ Aufstehen (“Stand Up”) was a left-wing political organization launched in Germany in 2018 by Sahra Wagenknecht after her departure from the Left Party (Die Linke). Following that, in 2023, Wagenknecht established Bündnis Sahra Wagenknecht (BSW (first as a political association, and then as a political party)

 5) Final point, concerning the integration of different type of issues (such as valence and position issues), I updated that part of the paper in the following way, emphasizing how digital crowdsourcing (especially at an initial stage) may be more suited for valence issues): Attempting to crowdsource an entire party program often proves unfeasible and results in unmet expectations. A more effective strategy is to proceed incrementally, beginning with less contentious, broadly supported topics, such as a social democratic party initiating a consultation on raising the minimum wage before addressing more polarizing issues like immigration. This is the case for valence issues, namely those topics on which there is broad cross-cutting agreement about the desired objective, but disagreement over which political actor is most competent and trustworthy to achieve it (Stokes, 1963). Unlike position issues (where competing policy preferences structure political conflict) valence issues are less likely to produce internal divisions. For this reason, initiating a crowdsourcing process on valence issues may allow a party to meaningfully incorporate citizens’ input while minimizing the risk of undermining its policy stances. Crucially, managing expectations is vital, as overly ambitious promises are likely to be disappointed, thereby undermining both the immediate initiative and the party's broader credibility for future participatory initiatives.

# Reviewer report: Reviewer response for version 1

**DOI:** 10.21956/openreseurope.23943.r66996

**Published:** 2026-01-16

**Authors:** Paolo Gerbaudo

**Affiliations:** 1 Department of Political Science and Administration, Complutense University of Madrid, Madrid, Spain

This is a thoughtful article which makes an innovative intervention in a growing area of scholarship about political parties: new forms of intra-party democracy and digital media. Specifically the focus is on practices of crowd-sourcing, as bottom-up channels by means of which poltical parties may be able to tap into people's knowledge and preferences,  thus overcoming some of the distance from the citizenry which currently results in very low approval rates. What is proposed is a theoretical discussion of how digital crowdsourcing may provide a way to move out some of the present impasses, also seen in recent political phenomena, that while integrating digtal media and deliberative practices are caught in self-defeating contradictions. I find the reconstruction of the debate clear and knowledgeable, and most importantly the typology of different digital crowd-sourcing practices very useful for analytical and orientational purposes. Similarly useful is the delineation of different challenges which party organisers would need to take into account in order to make the most of digital crowd-sourcing. I think that give the nature of the article as an exploratory contributon, there is no need for empirical support, besides the few examples that are already cited and that a more robust empirical analysis may be best suited to ensuing contributions within this research agenda. Otherwise, the article is very well structured, convincingly argued, and well written, and it seems to me to be ready for publication as it stands. One possible criticism would be that the conclusion as it is very short and one may have done without it, perhaps expanding a bit more the discussion part and turning it into a discussion and conclusion section. But more substantially I do not see the need for any significant revision as the article stands already well on its feet and makes a useful contribution to debates.

If applicable, is the statistical analysis and its interpretation appropriate?

Not applicable

Is the study design appropriate and is the work technically sound?

Yes

Is the work clearly and accurately presented and does it engage with the current literature?

Yes

Are the conclusions drawn adequately supported by the results?

Yes

Are sufficient details of methods and analysis provided to allow replication by others?

Not applicable

Are all the source data and materials underlying the results available?

Yes

Reviewer Expertise:

Political Science; Political Communication; Cultural Sociology

I confirm that I have read this submission and believe that I have an appropriate level of expertise to confirm that it is of an acceptable scientific standard.

# Response

**Published:** 2026-03-06

**Authors:** Francseco Nasi

**Affiliations:** Political and Social Sciences, Universita degli Studi di Bologna Dipartimento di Scienze Politiche e Sociali, Bologna, Emilia-Romagna, Italy

*With regard to Reviewer 1’s comment on the conclusion, I have expanded this section by revisiting several reflections related to party change and parties’ primary goals, so that the conclusion also offers a substantive contribution rather than merely summarizing the arguments presented earlier. The revised conclusion is as follows:* *“In this paper, I sought to connect research on digital crowdsourcing with the debate on intra-party democracy, deliberative democratic innovations and digital platforms. I argued that digital crowdsourcing may be better suited to advancing internal democracy than deliberative processes or comprehensive digital platforms. However, these instruments should not be viewed in opposition to one another. Depending on its political culture and priorities, each party should determine its own balance among different participatory strategies. Digital crowdsourcing is not a definitive solution but rather one additional tool in a broader repertoire, carrying its own advantages and limitations.* *We should always remember how it is inherently challenging for political parties to adopt such tools. This reluctance can be explained not only by internal power dynamics, but also by the very nature of political parties. Parties are conservative organizations (Panebianco, 1988). Their hierarchical structure serves the goal of maintaining clear policy positions. Evidence shows that support for democratic innovations is quite minoritarian, and “heavily related to anti-establishment parties, to left-wing parties and to parties with limites access to power” (Núñez, Close & Bedock, 2016, p. 1). Furthermore, even when their value and potential are acknowledged, participation and internal democracy rarely become primary goals.* *As Harmel and Janda (1994) argue, party change depends on parties’ primary goals. In turn, these goals may be shaped by a shift in the dominant coalition within the party or by institutional and organizational factors within the political system. More generally, however, parties’ primary goals tend to be outward-oriented, focused on vote-, office-, or policy-seeking. As a result, parties are often poorly equipped and incentivized to pause, reflect, and undertake substantial internal reforms. How, then, can an interest in a party’s own democratization become a priority? Most likely, to the extent that such a goal can go hand in hand with more immediate or pressing objectives, such as vote-seeking and policy-seeking. The adoption of participatory instruments such as crowdsourcing, therefore, should not be understood solely as a tool for strengthening internal democracy. It may also serve other goals, particularly improving policy development (policy-seeking) and projecting a more favorable image to the electorate (vote-seeking). By contrast, it may be less useful for parties that are primarily oriented toward office-seeking, insofar as it contributes little to their stability as governing partners.”*
